# Supplementary material for: Duration and dosing of systemic corticosteroids for acute exacerbation of COPD, protocol for a systematic review with meta-analysis of randomized trials and cohort studies
Source: PLoS One. 2023 Aug 31;18(8):e0290982. doi: 10.1371/journal.pone.0290982 (PMC10470871; doi:10.1371/journal.pone.0290982)
Supplement: S1 File — (PDF) [file pone.0290982.s002.pdf]

MEDLINE search strategy example:

1. exp Pulmonary Disease, Chronic Obstructive/
2. COPD.mp.
3. chronic obstructive pulmonary disease.mp.
4. exp Bronchitis, Chronic/
5. chronic bronchitis.mp.
6. exp Emphysema/
7. emphysema.mp.
8. 1 or 2 or 3 or 4 or 5 or 6 or 7
9. exp Steroids/
10. steroid*.mp.
11. exp Adrenal Cortex Hormones/
12. corticosteroid.mp.
13. exp Glucocorticoids/
14. glucocorticoid*.mp.
15. exp Beclomethasone/
16. beclomethasone.mp.
17. exp Betamethasone/
18. betamethasone.mp.
19. exp Fluticasone/
20. fluticasone.mp.
21. exp Cortisone/
22. cortisone.mp.
23. exp Dexamethasone/
24. dexamethasone.mp.
25. exp Hydrocortisone/
26. hydrocortisone.mp.
27. exp Prednisolone/
28. prednisolone.mp.
29. exp Prednisone/
30. prednisone.mp.
31. exp Methylprednisolone/
32. methylprednisolone.mp.
33. methylprednisone.mp.
34. exp Triamcinolone/
35. triamcinolone.mp.
36. 9 or 10 or 11 or 12 or 13 or 14 or 15 or 16 or 17 or 18 or 19 or 20 or 21 or 22 or 23 or 24 or 25 or 26 or 27 or 28 or 29 or 30 or 31 or 32 or 33 or 34 or 35
37. exp Disease Progression/

38. exacerb*.mp.
39. attack*.mp.
40. 37 or 38 or 39
41. 8 and 40
42. 36 and 41
