# Supplementary material for: Duration and dosing of systemic corticosteroids for acute exacerbation of COPD, protocol for a systematic review with meta-analysis of randomized trials and cohort studies
Source: PLoS One. 2023 Aug 31;18(8):e0290982. doi: 10.1371/journal.pone.0290982 (PMC10470871; doi:10.1371/journal.pone.0290982)
Supplement: Attachment — Submitted filename: Response to editor and reviewer.docx [file pone.0290982.s003.docx]

Dear editor and reviewers,

I have attached the newly revised version of our manuscript titled “Duration and dosing of systemic corticosteroids for acute exacerbation of COPD, protocol for a systematic review with meta-analysis of randomized trials and cohort studies” for publication at PLOS One. You will also find in the same submission a marked-up copy of the manuscript as well as an unmarked version of the revised paper.

We would like to give thanks for both the editor and the reviewers for their insightful comment. We have addressed them in our manuscript. The detailed changes are outlined in the paragraphs below as well as in the marked-up version of the manuscript. We would like to mention that we were not able to provide the minimal data set underlying the results, as this is a protocol paper and no minimal data set exist.

We would like to thank reviewer #1’s comment, and for taking their time in looking over the manuscript.

We have addressed the issue raised by reviewer #2 in our manuscript. We have added sentences in the materials and methods section (type of participants and data item sub-section) on extracting data regarding the comorbidities of these patients as well as the side effects, such as hypertension, diabetes, and heart failure. Additionally, we have added comments on extracting side effects post-corticosteroid administration. These comorbidities are common among COPD patients, and we thank the reviewer for pointing that out.

We have also addressed reviewer #3 / the editor’s comments on the protocol. After further consultation with an internal medicine specialist, we have now clearly defined the dosage cut-offs as well as refined our definition for short vs long duration corticosteroid administration. Short duration is now defined as ≤ 7 days whereas long duration is > 7 days. For cumulative dosage, low is defined as ≤ 300mg total and high is defined as > 300mg total, based on our preliminary readings of prior studies. These will be reflected in our final analysis, and we give our thanks to the editor.

Sincerely and thank you.

Winston Hou, corresponding author.
